# Mind that gap: A national survey of school-based approaches for improving student well-being

**DOI:** 10.1192/j.eurpsy.2023.1516

**Published:** 2023-07-19

**Authors:** H. Thabrew

**Affiliations:** Department of Psychological Medicine, University of Auckland, Auckland, New Zealand

## Abstract

**Introduction:**

Student well-being is an area of increasing interest for schools around the world. However, the extent to which school-based well-being and mental health interventions are currently being delivered by different schools has not previously been explored in many countries, including New Zealand.

**Objectives:**

This survey of a nationally-representative sample of schools was undertaken to identify: what well-being and mental health interventions were being used by primary (elementary) and secondary (high) schools and what gaps exist between current practice and the evidence-base.

**Methods:**

Forty staff from 37 (22 primary, 13 secondary and 2 composite) schools across New Zealand participated in semi-structured interviews. Data was analysed for key themes and subthemes using Braun and Clarke’s method.

**Results:**

Seven key themes were identified: 1) staff awareness and enthusiasm about student well-being and mental health; 2) existence of specific interventions to support student well-being and mental health; 3) support for government-sponsored programmes; 4) limitations of existing programmes; 5) drivers of new interventions; 6) barriers to implementation; and 7) suggestions for future interventions and their implementation.

**Conclusions:**

Despite enthusiasm from educators for interventions with which to improve student well-being, there is a gap between intention and activity. Students are receiving primarily non-evidence based interventions in a variable manner due to staff and cost-related barriers. We suggest ways for health and education providers in New Zealand and elsewhere to address these issues, as well as avenues for further research.

**Disclosure of Interest:**

None Declared

